# Compressive Sensing for Tomographic Imaging of a Target with a Narrowband Bistatic Radar

**DOI:** 10.3390/s19245515

**Published:** 2019-12-13

**Authors:** Ngoc Hung Nguyen, Paul Berry, Hai-Tan Tran

**Affiliations:** 1 Maritime Division, Defence Science and Technology Group, Edinburgh 5111, SA, Australia; 2 Intelligence, Surveillance and Space Division, Defence Science and Technology Group, Edinburgh 5111, SA, Australia; paul.berry@dst.defence.gov.au (P.B.); haitan.tran@dst.defence.gov.au (H.-T.T.)

**Keywords:** radar tomography, compressive sensing, sparse reconstruction, bistatic radar, radar imaging, parameter-refined orthogonal matching pursuit (PROMP), orthogonal matching pursuit (OMP), *k*-space tomography, narrowband radar, off-grid compressive sensing

## Abstract

This paper introduces a new approach to bistatic radar tomographic imaging based on the concept of compressive sensing and sparse reconstruction. The field of compressive sensing has established a mathematical framework which guarantees sparse solutions for under-determined linear inverse problems. In this paper, we present a new formulation for the bistatic radar tomography problem based on sparse inversion, moving away from the conventional *k*-space tomography approach. The proposed sparse inversion approach allows high-quality images of the target to be obtained from limited narrowband radar data. In particular, we exploit the use of the parameter-refined orthogonal matching pursuit (PROMP) algorithm to obtain a sparse solution for the sparse-based tomography formulation. A key important feature of the PROMP algorithm is that it is capable of tackling the dictionary mismatch problem arising from off-grid scatterers by perturbing the dictionary atoms and allowing them to go off the grid. Performance evaluation studies involving both simulated and real data are presented to demonstrate the performance advantage of the proposed sparsity-based tomography method over the conventional *k*-space tomography method.

## 1. Introduction

Radar imaging has received much attention for several decades, having a wide range of applications in both civilian and military domains [1,2,3]. In principle, to obtain high-resolution radar images, a wide bandwidth of radar waveform is required for a fine resolution in the range direction, while a large antenna aperture is required for a fine resolution in the cross-range direction. To overcome the physical constraints of the radar aperture size, a synthesized aperture with a much larger size can be formed by exploiting the relative motion between the radar and target. This is, in fact, the main idea behind the synthetic aperture radar (SAR) and inverse SAR (ISAR) [1]. In recent years, there has been an increasing demand on the radio-frequency (RF) electromagnetic spectrum due to rapid advances in radar and communications, and the radar has to compete for spectrums with many different services, including radio and television broadcasting, communications, and radio-navigation [4]. As a result, the constraints on spectrum availability may present severe limits on signal bandwidth, prompting the need for high-resolution imaging techniques using narrowband radars.

As a consequence, Doppler tomography has been considered for narrowband radar imaging [5,6,7,8,9,10,11,12], which is also called “Doppler radar tomography” (DRT). The main idea of DRT is to utilize the information given by the Doppler frequencies induced from the relative radar-target rotational motion to construct an image of the target, which can be conveniently formulated in the slow-time *k*-space [8]. Imaging can also be formulated in the more traditional fast-time *k*-space, the support for which is created by sweeping out the complex samples of the received signal in the angular direction. For a particular transmit frequency, the complex samples for all available aspect angles form a circular arc in the spatial frequency space. The traditional range-Doppler ISAR imaging can be considered as a special case of this fast-time *k*-space technique when a wideband signal is available and the total rotation angle is small enough that the support region can be approximated as being rectangular. The inversion process for image formation has evolved from traditional tools, such as filtered back projection, to the more modern non-uniform fast Fourier transform (NUFFT). The *k*-space radar tomography was also considered in bistatic settings [13,14]. The bistatic radar offers several advantages over a monostatic radar, including higher performance against stealth targets, less vulnerability to jamming, and its covertness.

The main objective of this paper is to present a new tomographic imaging technique for a narrowband bistatic radar based on the framework of compressive sensing and sparse reconstruction. The field of compressive sensing has established a mathematical framework which guarantees sparse solutions for underdetermined linear inverse problems that occur across numerous engineering and mathematical science fields. In particular, this framework has found applications in various radar imaging problems, ranging from moving target indication, ISAR imaging, coherence imaging, multichannel imaging, micro-Doppler imaging, to through-the-wall radar imaging (see, e.g., [15,16,17,18,19,20,21,22,23,24,25,26]). The key contributions of this paper are summarized as follows.

- A new formulation for radar tomography based on sparse inversion is introduced. The main idea is to construct a dictionary of signal prototypes by discretizing the illuminated scene of interest into a grid of discrete points. In this formulation, the received radar signal vector becomes linear to the unknown reflection vector to be estimated. This effectively casts the radar tomography problem under consideration to a sparse linear inverse problem, given that the illuminated scene of interest only contains a small number of dominant scatterers, as is often the case in practice. Such a formulation allows a high-quality image of the target to be obtained under the compressive sensing framework.
- A technical challenge for the tomography formulation based on sparse inversion is the dictionary mismatch problem, resulting from the fact that the true scatterers almost always do not coincide exactly with the dictionary grid. This dictionary mismatch problem has been known in the literature to significantly degrade the performance of conventional sparse reconstruction techniques [27,28]. To overcome this problem, we tried exploiting the use of the parameter-refined orthogonal matching pursuit (PROMP) algorithm [29] to solve the sparsity-based tomographic formulation. Compared to other conventional sparse reconstruction techniques, like the orthogonal matching pursuit (OMP) and convex optimization (see e.g., [30,31], and the references therein), PROMP has the advantage of being capable of dealing with the dictionary mismatch arising from off-grid scatterers by perturbing the dictionary atoms and allowing them to go off the grid. PROMP belongs to the greedy pursuit family which identifies the support of the solution in an iterative manner based on the level of correlation between the input data and the dictionary atoms. As a result, PROMP is computationally efficient and thus suitable for real-time operation.
- Performance evaluation studies involving both simulated and real data are presented to demonstrate the superior performance of the proposed sparsity-based tomography method over the conventional *k*-space tomography technique.

The remainder of this paper is organized as follows. Section 2 describes the signal model for bistatic radar tomography. Section 3 formulates bistatic radar tomographic imaging as a sparse inversion problem. Section 4 derives the sparse solution based on the PROMP algorithm. Performance studies with simulated and real data are presented in Section 5. The paper ends in Section 6 with some concluding remarks.

## 2. Signal Model

**Figure 1:**
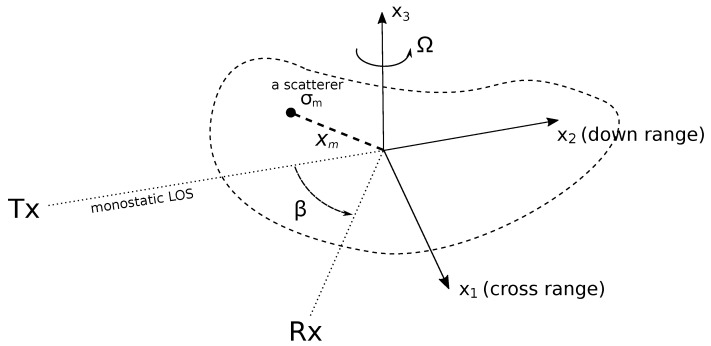
The radar-target geometry of the considered bistatic radar tomographic imaging problem.

Figure 1 shows the geometry for the problem of bistatic radar tomographic imaging under consideration. The transmitter Tx and receiver Rx are located in the far field of the target of interest. The bistatic angle between the transmitter and receiver with respect to the target is denoted as β. The transmitter is narrowband, transmitting a continuous waveform at a single frequency *f* (i.e., the wavelength is λ=c/f, where *c* is the speed of signal propagation). A local target coordinate frame $T(x_{1},x_{2},x_{3})$, which is fixed and rotated with the target, is chosen as the reference frame. Here, both the transmitter and receiver lie on the image plane $X(x_{1},x_{2})$ of the target, and the origin of the frame T is placed at the target rotation centre. It is also assumed that the target rotational speed Ω is constant over the coherent processing interval (CPI) and is accurately estimated a priori.

The receiver takes one complex-valued scattered signal sample for each rotation angle $\theta_{n}=\Omega t_{n}$ of the target (with respect to the axis $x_{2}$ of the frame T) at time $t_{n}$. The expression of the scattered signal sample collected at time $t_{n}$ is given by [13,14]

(1) $$s\left(t_{n}\right)=\int_{x_{1}}\int_{x_{2}}\sigma \left(x\right)\exp\left\{-j\frac{2\pi }{c}f\left[R\left(t_{n}\right)+2\cos\left(\frac{1}{2}\left|\beta \right|\right)\;x\cdot u\left(t_{n}\right)\right]\right\}dx_{1}dx_{2},$$

where $x={\left[x_{1},x_{2}\right]}^{T}$, *R* is the total bistatic range between the target centre (or focus point) and the transmitter and receiver, and σ(x) is the scatterer reflectivity distribution projected onto the image plane. Note that the total bistatic range *R* is, in general, a function of time $t_{n}$ because of the target translational motion. In (1), $u={\left[u_{1},u_{2}\right]}^{T}$ denotes the unit vector along the bisector of the bistatic angle β. It is also noted that the radar tomographic imaging problem under interest is considered in the rotating local frame T, and the signal model (1) has already taken into account the rotational motion of the target by the rotation of the unit vector $u(t_{n})$ relative to T.

Since $x_{1}$ and $x_{2}$ in the image domain are discrete variables in practical radar imaging applications, the reflectivity function σ(x) is commonly discretized over the image plane $X(x_{1},x_{2})$ onto a grid of points $x_{m}$ for m∈{1,…,M}, as

(2) $$\sigma \left(x\right)=\sum_{m=1}^{M}\sigma_{m}\delta \left(x-x_{m}\right).$$

Substituting (2) into (1) and assuming that translational motion compensation is accurately accomplished by additional pre-processing, we obtain

(3) $$\begin{array}{cc} s\left(t_{n}\right)=\sum_{m=1}^{M} & \;\sigma_{m}\exp\left\{-j\frac{4\pi }{c}f\cos\left(\frac{1}{2}\left|\beta \right|\right)\;x_{m}\cdot u\left(t_{n}\right)\right\}. \end{array}$$

A compact vector form of (3) is given by

(4) s=Φσ,

where

(5) $$\begin{array}{cc} s & ={\left[s\left(t_{1}\right),\ldots ,s\left(t_{N}\right)\right]}^{T} \end{array}$$

(6) $$\begin{array}{cc} \sigma & ={\left[\sigma_{1},\ldots ,\sigma_{M}\right]}^{T} \end{array}$$

and

(7a) $$\begin{array}{cc} \Phi & =[\phi \left(x_{1}\right),\ldots ,\phi \left(x_{M}\right)] \end{array}$$

(7b) $$\begin{array}{cc} \phi (x_{m}) & ={\left[\phi \left(x_{m},t_{1}\right),\ldots ,\phi \left(x_{m},t_{N}\right)\right]}^{T} \end{array}$$

(7c) $$\begin{array}{cc} \phi (x_{m},t_{n}) & =\exp\left\{-j\frac{4\pi }{c}f\cos\left(\frac{1}{2}\left|\beta \right|\right)\;x_{m}\cdot u\left(t_{n}\right)\right\}. \end{array}$$

In practice, where noise is presented, the radar received signal becomes

(8) $$\tilde{s}=s+n=\Phi \sigma +n,$$

where $n={\left[n\left(t_{1}\right),\ldots ,n\left(t_{N}\right)\right]}^{T}$, with $n(t_{n})$ denoting the complex-valued noise term at time $t_{n}$.

## 3. Sparse Inversion Formulation of Bistatic Radar Tomography

The objective of any target imaging problem is to construct a spatial reflectivity map of the target from the backscattered radar signal. Specifically, the ultimate objective is to estimate the unknown reflection vector σ from the noisy received signal vector $\tilde{s}$ by solving (8). Since the number of signal samples received, as often is the case in practice, is much smaller than the number of grid points in the reflectivity map (i.e., N≪M), solving (8) is essentially an underdetermined linear inverse problem which requires additional regularization constraints to obtain meaningful solutions.

Typical target images captured by microwave radar signals has been known in the literature to contain a few dominant scattering centers (see, e.g., [15,16,17,19,20,21,22,23]). As a result, the reflection vector σ only has a small number of non-zero elements, thus enjoying a sparse characteristic. Such a sparse characteristic of σ can be utilized as a regularizing constraint to solve the underdetermined inverse problem (8), that is,

(9) $$\mathrm{find}\;\mathrm{sparse}\;\sigma \;\mathrm{such}\;\mathrm{that}\;\tilde{s}\approx \Phi \sigma .$$

This sparse inversion problem can be effectively solved under the compressive sensing framework using sparse reconstruction algorithms. Note that in the compressive sensing context, the matrix **Φ** is commonly referred to as the dictionary, and the columns of **Φ** are called the atoms, each representing the theoretical scattered signal component of a hypothetical scatterer residing on a grid point of the reflectivity map.

Compressive sensing and sparse reconstruction have been extensively studied in the last two decades, with various techniques proposed. Comprehensive surveys of the state-of-the-art on this topic can be found in [22,23,30,31]. The objective of this paper is to apply the sparse reconstruction approach to the bistatic radar tomographic imaging problem.

The main challenge for this work is that the true scatterers constituting the target do not coincide exactly with the grid which is used to construct the dictionary, leading to dictionary mismatch problems which in turn significantly degrade the performance of conventional sparse reconstruction techniques [27,28]. Several methods have been presented in the literature to address the off-grid dictionary mismatch problems based on the ideas of joint-sparse recovery [32], dictionary perturbation [33,34], sparse Bayesian learning [35,36], and parameter perturbation [29,37]. In this paper, we will exploit the use of the PROMP method [29,37], that is, a parameter perturbation method, to solve the sparse inversion problem (9). The main motivations of using PROMP are twofold. Firstly, PROMP is capable of tackling the dictionary mismatch problem by perturbing the dictionary atoms and allowing them to go off the grid. Secondly, PROMP is computationally efficient and thus suitable for real-time operation because it belongs to the greedy pursuit family which identifies the support of the solution in an iterative manner based on the level of correlation between the input data and the dictionary atoms.

## 4. Parameter-Refined Orthogonal Matching Pursuit

Table 1 summarizes the overall structure of the PROMP algorithm. As a variant of the greedy pursuit technique, PROMP solves the sparse inversion problem (9) by identifying the support of σ in an iterative greedy manner. In particular, it starts with an empty support set $\Lambda^{\left[0\right]}=\emptyset$ and sets the signal $\tilde{s}$ as the initial signal residual $r^{\left[0\right]}$. Like other greedy techniques, one column of Φ (corresponding to one atom of the dictionary) that produces the largest correlation with the current signal residual $r^{\left[i\right]}$ is chosen and added to the support set $\Lambda^{\left[i\right]}$ in each iteration. However, a unique feature of PROMP is that it allows the dictionary atoms to go off the grid by perturbing their parameters, thus it can overcome the off-grid dictionary mismatch problem. Specifically, the updated step of PROMP, as different to that of other greedy pursuit techniques, not only estimates the coefficients $\sigma_{k}^{\left[i\right]}$ but also determines the positions $x_{k}^{\left[i\right]}={\left[x_{1,k}^{\left[i\right]},x_{2,k}^{\left[i\right]}\right]}^{T}$, k=1,…,i, of the scatterers associated with the current support set $\Lambda^{\left[i\right]}$ via the least-square sense as

(10) $$\begin{array}{c} \;{\left\{{\hat{\sigma }}_{k}^{\left[i\right]},{\hat{x}}_{k}^{\left[i\right]}\right\}}_{k=1,\ldots ,i}=arg min∥\tilde{s}-\sum_{k=1}^{i}\sigma_{k}^{\left[i\right]}\phi \left(x_{k}^{\left[i\right]}\right){∥}_{2}\\ \mathrm{subject}\;\mathrm{to}\\ \;∥{\hat{x}}_{1,k}^{\left[i\right]}-{\bar{x}}_{1,k}^{\left[i\right]}∥\leq \zeta \;\mathrm{and}\;∥{\hat{x}}_{2,k}^{\left[i\right]}-{\bar{x}}_{2,k}^{\left[i\right]}∥\leq \zeta , \end{array}$$

which is in fact a nonlinear least-square (NLS) estimation problem. Here, ${\bar{x}}_{k}^{\left[i\right]}={\left[{\bar{x}}_{1,k}^{\left[i\right]},{\bar{x}}_{2,k}^{\left[i\right]}\right]}^{T}$, k=1,…,i are the positions of the dictionary atoms in the current support set $\Lambda^{\left[i\right]}$. Note that the constraint in (10) ensures the position estimate for each scatterer staying within the vicinity of the corresponding dictionary atom. A nominal resolution of λ/2 can be used to set the value of ζ.

Since the NLS problem in (10) does not admit a closed-form solution, in what follows we will derive an iterative solution based on the Gauss–Newton (GN) approach [38]. Note that the scatterer reflection coefficient is a complex-valued variable, while the scatterer position is a real-valued variable. For the sake of convenience, we transform (10) into a NLS problem purely in the real-valued domain. In particular, we re-express the cost function in (10) as

(11) $${∥\tilde{s}-\sum_{k=1}^{i}\sigma_{k}^{\left[i\right]}\phi \left(x_{k}^{\left[i\right]}\right)∥}_{2}={∥\left[\begin{array}{c} \mathrm{Real}\{\tilde{s}\}\\ \mathrm{Imag}\{\tilde{s}\} \end{array}\right]-\sum_{k=1}^{i}\left[\begin{array}{c} \mathrm{Real}\left\{\sigma_{k}^{\left[i\right]}\phi \left(x_{k}^{\left[i\right]}\right)\right\}\\ \mathrm{Imag}\left\{\sigma_{k}^{\left[i\right]}\phi \left(x_{k}^{\left[i\right]}\right)\right\}, \end{array}\right]∥}_{2}$$

where explicit expressions of $\mathrm{Real}\left\{\sigma_{k}^{\left[i\right]}\phi \left(x_{k}^{\left[i\right]}\right)\right\}$ and $\mathrm{Imag}\left\{\sigma_{k}^{\left[i\right]}\phi \left(x_{k}^{\left[i\right]}\right)\right\}$ are given by

(12a) $$\begin{array}{cc} \mathrm{Real}\left\{\sigma_{k}^{\left[i\right]}\phi \left(x_{k}^{\left[i\right]}\right)\right\} & ={\left[\ldots ,\left(\mathrm{Real}\left\{\sigma_{k}^{\left[i\right]}\right\}\cos\theta_{k}^{\left[i\right]}\left(t_{n}\right)-\mathrm{Imag}\left\{\sigma_{k}^{\left[i\right]}\right\}\sin\theta_{k}^{\left[i\right]}\left(t_{n}\right)\right),\ldots \right]}_{n=1,\ldots ,N}^{T}, \end{array}$$

(12b) $$\begin{array}{cc} \mathrm{Imag}\left\{\sigma_{k}^{\left[i\right]}\phi \left(x_{k}^{\left[i\right]}\right)\right\} & ={\left[\ldots ,\left(\mathrm{Real}\left\{\sigma_{k}^{\left[i\right]}\right\}\sin\theta_{k}^{\left[i\right]}\left(t_{n}\right)+\mathrm{Imag}\left\{\sigma_{k}^{\left[i\right]}\right\}\cos\theta_{k}^{\left[i\right]}\left(t_{n}\right)\right),\ldots \right]}_{n=1,\ldots ,N}^{T}, \end{array}$$

and

(13) $$\theta_{k}^{\left[i\right]}\left(t_{n}\right)=-\frac{4\pi f}{c}\cos\left(\frac{\left|\beta \right|}{2}\right)\left(x_{1,k}^{\left[i\right]}u_{1}\left(t_{n}\right)+x_{2,k}^{\left[i\right]}u_{2}\left(t_{n}\right)\right).$$

Now we define

(14) $$\tilde{z}=\left[\begin{array}{c} \mathrm{Real}\{\tilde{s}\}\\ \mathrm{Imag}\{\tilde{s}\} \end{array}\right],\;z=\sum_{k=1}^{i}\left[\begin{array}{c} \mathrm{Real}\left\{\sigma_{k}^{\left[i\right]}\phi \left(x_{k}^{\left[i\right]}\right)\right\}\\ \mathrm{Imag}\left\{\sigma_{k}^{\left[i\right]}\phi \left(x_{k}^{\left[i\right]}\right)\right\} \end{array}\right]$$

and

(15) $$\begin{array}{c} \xi^{\left[i\right]}={\left[\xi_{1}^{[i]T},\ldots ,\xi_{i}^{[i]T}\right]}^{T}\mathrm{with}\;\xi_{k}^{\left[i\right]}={\left[\sigma_{R,k}^{\left[i\right]},\sigma_{I,k}^{\left[i\right]},x_{1,k}^{\left[i\right]},x_{2,k}^{\left[i\right]}\right]}^{T}. \end{array}$$

Here, $\sigma_{R,k}^{\left[i\right]}=\mathrm{Real}\left\{\sigma_{k}^{\left[i\right]}\right\},\sigma_{I,k}^{\left[i\right]}=\mathrm{Imag}\left\{\sigma_{k}^{\left[i\right]}\right\}$. Noting that z is a function of $\xi^{\left[i\right]}$, (10) is equivalent to

(16) $${\hat{\xi }}^{\left[i\right]}=\underset{\xi^{\left[i\right]}}{arg min}{∥\tilde{z}-z\left(\xi^{\left[i\right]}\right)∥}_{2}.$$

This is a NLS problem solely in the real-valued domain, and its solution can be obtained via the following GN iteration [38]

(17) $${\hat{\xi }}^{\left[i\right]}\left(h+1\right)={\hat{\xi }}^{\left[i\right]}\left(h\right)+{\left(\Gamma^{T}\left(h\right)\Gamma \left(h\right)\right)}^{-1}\Gamma^{T}\left(h\right)\left(\tilde{z}-z\left({\hat{\xi }}^{\left[i\right]}\left(h\right)\right)\right)$$

for h=0,1,…, where $\Gamma \left(h\right)=\Gamma \left({\hat{\xi }}^{\left[i\right]}\left(h\right)\right)$ is the Jacobian matrix of z with respect to $\xi^{\left[i\right]}$ evaluated at $\xi^{\left[i\right]}={\hat{\xi }}^{\left[i\right]}\left(h\right)$ and $z\left(\hat{\xi }\right)$ is an estimate of z calculated at $\xi^{\left[i\right]}={\hat{\xi }}^{\left[i\right]}\left(h\right)$.

The expression of the Jacobian matrix $\Gamma (\xi^{\left[i\right]})$ is given by

(18a) $$\begin{array}{cc} \Gamma & ={\left[\ldots ,\Gamma_{k},\ldots \right]}_{k=1,\ldots ,i} \end{array}$$

(18b) $$\begin{array}{cc} \Gamma_{k} & =\left[\begin{array}{cccc} \Gamma_{k}^{\left(1\right)} & \Gamma_{k}^{\left(2\right)} & \Gamma_{k}^{\left(3\right)} & \Gamma_{k}^{\left(4\right)}\\ \Gamma_{k}^{\left(5\right)} & \Gamma_{k}^{\left(6\right)} & \Gamma_{k}^{\left(7\right)} & \Gamma_{k}^{\left(8\right)} \end{array}\right] \end{array}$$

(18c) $$\begin{array}{cc} \Gamma_{k}^{\left(1\right)} & ={\left[\ldots ,\cos\theta_{k}^{\left[i\right]}\left(t_{n}\right),\ldots \right]}_{n=1,\ldots ,N}^{T} \end{array}$$

(18d) $$\begin{array}{cc} \Gamma_{k}^{\left(5\right)} & ={\left[\ldots ,\sin\theta_{k}^{\left[i\right]}\left(t_{n}\right),\ldots \right]}_{n=1,\ldots ,N}^{T} \end{array}$$

(18e) $$\begin{array}{cc} \Gamma_{k}^{\left(2\right)} & ={\left[\ldots ,-\sin\theta_{k}^{\left[i\right]}\left(t_{n}\right),\ldots \right]}_{n=1,\ldots ,N}^{T} \end{array}$$

(18f) $$\begin{array}{cc} \Gamma_{k}^{\left(6\right)} & ={\left[\ldots ,\cos\theta_{k}^{\left[i\right]}\left(t_{n}\right),\ldots \right]}_{n=1,\ldots ,N}^{T} \end{array}$$

(18g) $$\begin{array}{cc} \Gamma_{k}^{\left(3\right)} & ={\left[\ldots ,\frac{4\pi f}{c}\cos\left(\frac{\left|\beta \right|}{2}\right)u_{1}\left(t_{n}\right)\left(\sigma_{R,k}^{\left[i\right]}\sin\theta_{k}^{\left[i\right]}\left(t_{n}\right)+\sigma_{I,k}^{\left[i\right]}\cos\theta_{k}^{\left[i\right]}\left(t_{n}\right)\right),\ldots \right]}_{n=1,\ldots ,N}^{T} \end{array}$$

(18h) $$\begin{array}{cc} \Gamma_{k}^{\left(7\right)} & ={\left[\ldots ,\frac{4\pi f}{c}\cos\left(\frac{\left|\beta \right|}{2}\right)u_{1}\left(t_{n}\right)\left(\;-\;\sigma_{R,k}^{\left[i\right]}\cos\theta_{k}^{\left[i\right]}\left(t_{n}\right)+\sigma_{I,k}^{\left[i\right]}\sin\theta_{k}^{\left[i\right]}\left(t_{n}\right)\right),\ldots \right]}_{n=1,\ldots ,N}^{T} \end{array}$$

(18i) $$\begin{array}{cc} \Gamma_{k}^{\left(4\right)} & ={\left[\ldots ,\frac{4\pi f}{c}\cos\left(\frac{\left|\beta \right|}{2}\right)u_{2}\left(t_{n}\right)\left(\sigma_{R,k}^{\left[i\right]}\sin\theta_{k}^{\left[i\right]}\left(t_{n}\right)+\sigma_{I,k}^{\left[i\right]}\cos\theta_{k}^{\left[i\right]}\left(t_{n}\right)\right),\ldots \right]}_{n=1,\ldots ,N}^{T} \end{array}$$

(18j) $$\begin{array}{cc} \Gamma_{k}^{\left(8\right)} & ={\left[\ldots ,\frac{4\pi f}{c}\cos\left(\frac{\left|\beta \right|}{2}\right)u_{2}\left(t_{n}\right)\left(\;-\;\sigma_{R,k}^{\left[i\right]}\cos\theta_{k}^{\left[i\right]}\left(t_{n}\right)+\sigma_{I,k}^{\left[i\right]}\sin\theta_{k}^{\left[i\right]}\left(t_{n}\right)\right),\ldots \right]}_{n=1,\ldots ,N}^{T}. \end{array}$$

The following decision logic is then applied at the end of each GN iteration to ensure the constraint in (10) is met:

(19) $$\begin{array}{cc} \; & \mathrm{if}\left({\hat{x}}_{1,k}^{\left[i\right]}\left(h+1\right)-{\bar{x}}_{1,k}^{\left[i\right]}\right)≷\pm \zeta \mathrm{set}{\hat{x}}_{1,k}^{\left[i\right]}\left(h+1\right)={\bar{x}}_{1,k}^{\left[i\right]}\pm \zeta ,\\ \; & \mathrm{if}\left({\hat{x}}_{2,k}^{\left[i\right]}\left(h+1\right)-{\bar{x}}_{2,k}^{\left[i\right]}\right)≷\pm \zeta \mathrm{set}{\hat{x}}_{2,k}^{\left[i\right]}\left(h+1\right)={\bar{x}}_{2,k}^{\left[i\right]}\pm \zeta . \end{array}$$

When the constraint is in effect, a re-estimation of the reflection coefficients is performed as

(20) $${\left[{\hat{\sigma }}_{1}^{\left[i\right]}\left(h+1\right),\ldots ,{\hat{\sigma }}_{i}^{\left[i\right]}\left(h+1\right)\right]}^{T}={\left(\Upsilon^{H}\Upsilon \right)}^{-1}\Upsilon^{H}\tilde{s}$$

with $\Upsilon =\left[\phi \left({\hat{x}}_{1}^{\left[i\right]}\left(h+1\right)\right),\ldots ,\phi \left({\hat{x}}_{i}^{\left[i\right]}\left(h+1\right)\right)\right]$. The GN iteration can be halted after a fixed number of iterations or if the $l_{2}$ norm of the updating term falls below a given threshold.

The GN iteration is initialized to the solution ${\hat{\xi }}^{\left[i-1\right]}$ obtained from the previous PROMP iteration i−1 and the newly selected atom:

(21) $${\hat{\xi }}^{\left[i\right]}\left(0\right)={\left[{\hat{\xi }}^{[i-1]T},\mathrm{Real}\left\{{\bar{\sigma }}_{j^{\left[i\right]}}\right\},\mathrm{Imag}\left\{{\bar{\sigma }}_{j^{\left[i\right]}}\right\},{\bar{x}}_{j^{\left[i\right]}}^{T}\right]}^{T}$$

where ${\bar{x}}_{j^{\left[i\right]}}$ is the position of the newly selected atom at index $j^{\left[i\right]}$ within the dictionary and ${\bar{\sigma }}_{j^{\left[i\right]}}={\left(\phi^{H}\left({\bar{x}}_{j^{\left[i\right]}}\right)\phi \left({\bar{x}}_{j^{\left[i\right]}}\right)\right)}^{-1}\phi^{H}\left({\bar{x}}_{j^{\left[i\right]}}\right)r^{\left[i-1\right]}$ is the corresponding initial coefficient estimate for this atom.

## 5. Results

In this section, we demonstrate the performance superiority of the proposed sparsity-based tomography method based on the PROMP algorithm over the conventional *k*-space tomography method via results using both simulated and real data. The result comparison also includes the performance of the OMP algorithm to illustrate the off-grid dictionary mismatch problem of the sparsity-based tomography formulation and to verify the effectiveness of PROMP in dealing with this issue.

### 5.1. Results with Simulated Data

**Figure 2:**
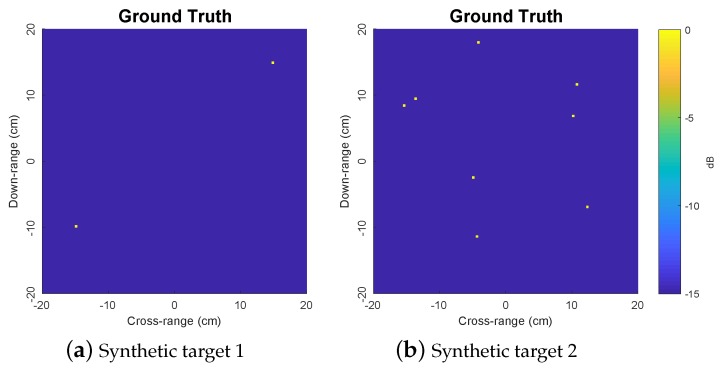
Ground truth images of two synthetic targets under consideration.

We consider two synthetic targets with two and eight scatterers, respectively, as depicted in Figure 2. In this simulation, the target rotational speed is set to Ω=37.70 rad/s and the signal frequency is set to f=9.96 GHz. The constraint value ζ is set to ζ=λ/2 for PROMP. The dictionary matrix is constructed using a regularly spaced grid in Cartesian coordinates with the grid step size of Δ=λ/5 on each *x*- and *y*-axis. The sampling frequency at the receiver is set to 2.16 kHz. PROMP and OMP iterations are halted if the signal residual reaches the noise level.

**Figure 3:**
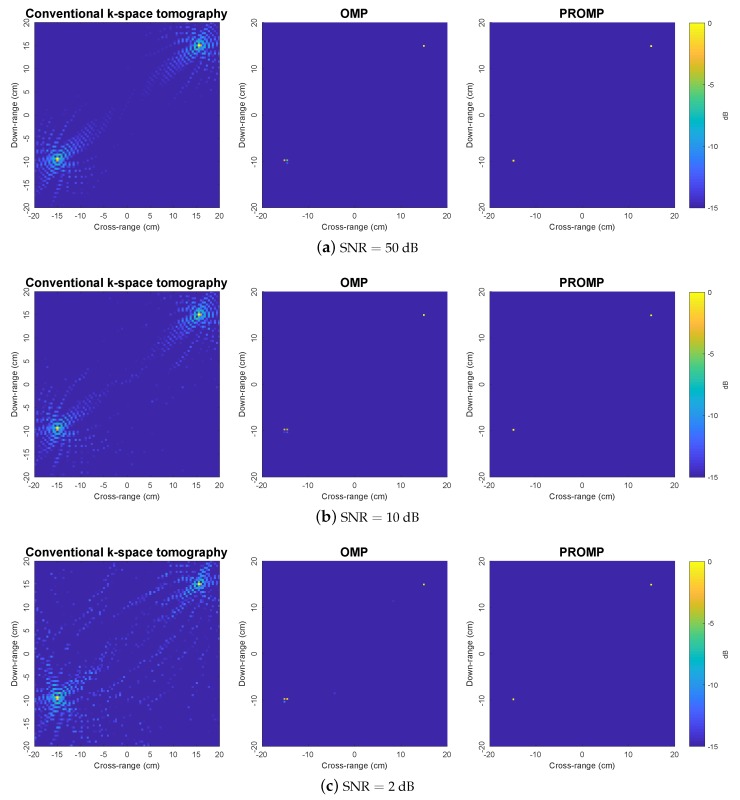
Reconstructed images obtained by the *k*-space, OMP and PROMP algorithms for synthetic target 1.

Figure 3 compares the reconstructed images for synthetic target 1 obtained by the *k*-space, OMP and PROMP algorithms for various noise levels. Here, the number of data samples is N=360 (i.e., the CPI approximately being one full rotation cycle of the target). Note that the SNR is defined by $\mathrm{SNR}=20\log_{10}(∥s∥/∥n∥)$. We observe that the *k*-space technique produces images with two main peaks corresponding to the true target scatterers. However, along with these two main peaks, the images obtained by the *k*-space technique also contain other sidelobes with many spurious peaks. Specifically, the higher the noise is, the poorer the performance of the *k*-space technique (i.e., yielding a larger numbers of spurious peaks). In contrast, such a problem associated with spurious peaks does not appear in the OMP and PROMP images, thus demonstrating the performance advantage of the sparsity-based tomography approach over the conventional *k*-space approach. It is observed that one of the scatterers is split into multiple peaks in the OMP images. This observation can be explained by the fact the OMP solution relies on the fixed dictionary which is built based on a grid of atoms while the true scatterers of the target do not coincide with this dictionary grid, thereby demonstrating the dictionary mismatch problem. On the other hand, by perturbing the dictionary atoms and allowing them to go off the grid, the PROMP algorithm can effectively overcome the dictionary mismatch problem by exhibiting a clean image with only two peaks corresponding to the true scatterers. More importantly, the locations of the peaks in the PROMP images almost exactly match the locations of the true scatterers.

**Figure 4:**
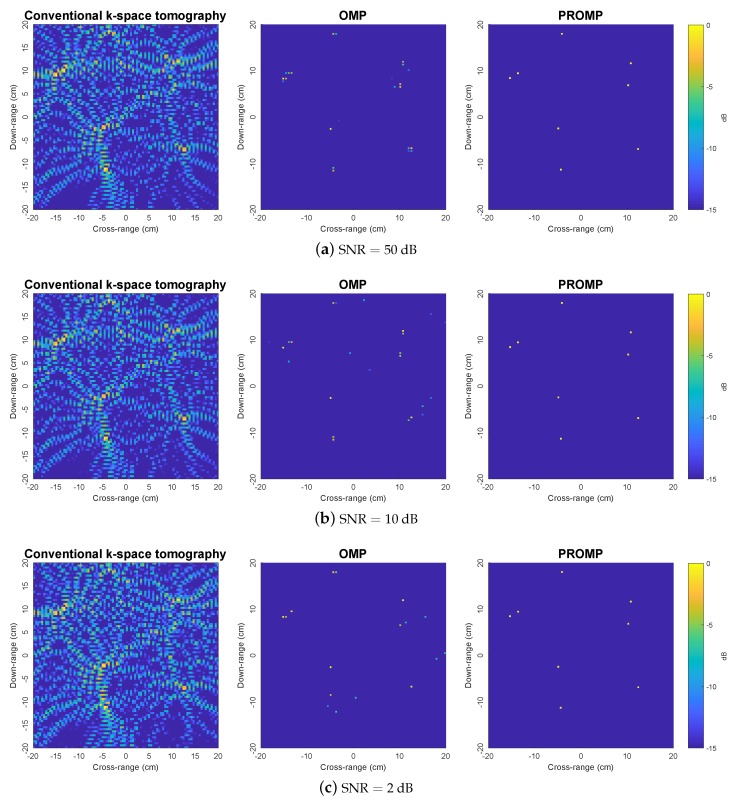
Reconstructed images obtained by the *k*-space, OMP and PROMP algorithms for synthetic target 2.

Figure 4 shows the results for synthetic target 2. This is a more challenging scenario because target 2 contains much more scatterers than target 1. We observer that the conventional *k*-space method is struggling to produce reliable image results because of the interaction between the sidelobes of different main peaks, especially in large noise scenarios. Such an interaction leads to some strong spurious peaks which have similar magnitudes to the correct peaks that correspond to the true scatterers, thus making the resulting images severely distorted. On the other hand, compared to the *k*-space method, OMP results in much more satisfactory images. However, the OMP performance is significantly affected by the dictionary mismatch problem arising from off-grid scatterers. As a result, the true scatterers are split into multiple peaks in the OMP images, and some spurious peaks also appear. In contrast, PROMP produces clean and clear images which are almost identical to the ground truth target image, even at large noise levels.

**Figure 5:**
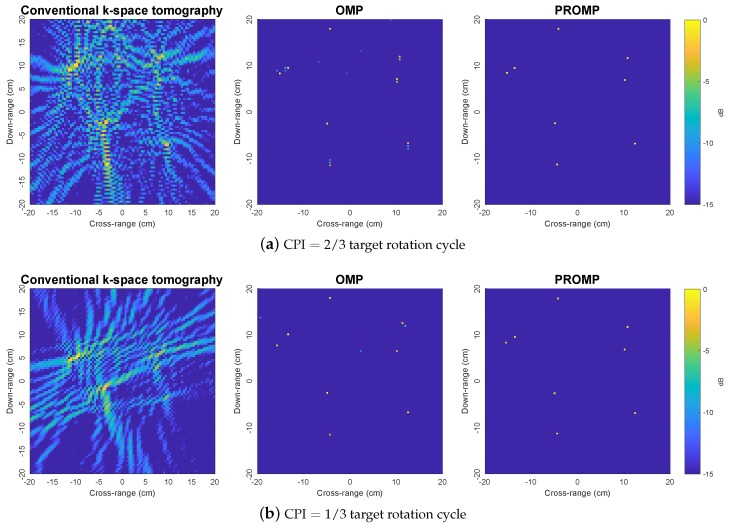
Reconstructed images obtained by the *k*-space, OMP and PROMP algorithms for synthetic target 2, given less data samples.

To further demonstrate the superior performance of PROMP, the reconstructed images obtained from less data samples (i.e., with CPI=2/3 and 1/3 target rotation cycle) are shown in Figure 5. With a limited number of data samples, the *k*-space method results in unsatisfactory images with incorrect peaks, while OMP and PROMP are observed to retain their good performance. In addition, similar to the observations in Figure 4, PROMP outperforms OMP and provides a better image of the target thanks to its ability to deal with off-grid scatterers.

Note that, to satisfy the sparsity condition, the number of dominant scatterers constituting the target must be sufficiently small compared to the number of grid points on the reflectivity map. The required sparsity level in general depends on several factors, including the number of data samples, the noise level, as well as the level of coherence between the atoms of the dictionary. In compressive sensing, the restricted isometry property and the mutual incoherence property establish theoretical connections between those factors required for the effectiveness of sparse reconstruction [23]. However, these analytical metrics are overly-conservative and do not reflect the average performance which is often of interest from the practical point of view [39].

**Figure 6:**
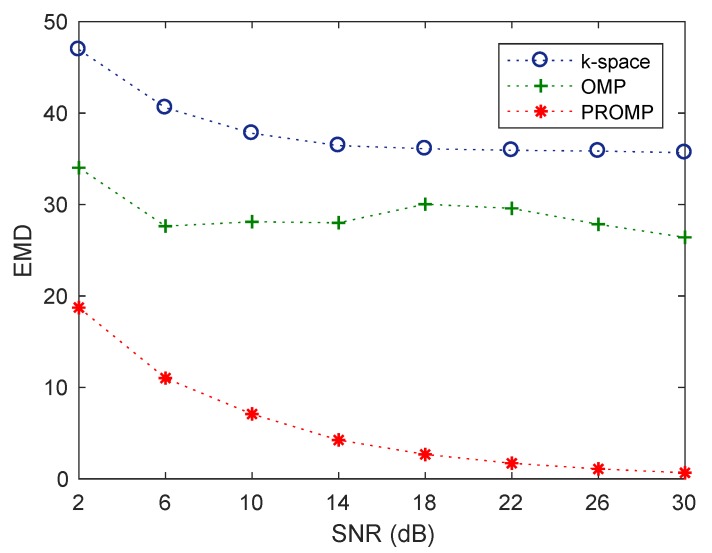
EMD performance of the *k*-space, OMP and PROMP algorithms versus SNR for synthetic target 2 and CPI=1 target rotation cycle.

We now compare the performance of the *k*-space, OMP, and PROMP methods using the earth mover’s distance (EMD) between the true and reconstructed images. EMD [40] is a widely-used metric to compare the similarity between different images. In principle, EMD is an estimate of the distance between two distributions which is equivalent to the minimal amount of work required for one distribution to be transformed to the other [40]. Figure 6 shows the EMD performance of the *k*-space, OMP, and PROMP methods, averaged from 1000 Monte Carlo runs, against various levels of SNR for the synthetic target 2 and CPI=1 target rotation cycle. We observe that the PROMP method exhibits an EMD much smaller than those of the *k*-space and OMP methods. This indicates that, from a statistical point of view, the image obtained by PROMP is much closer to the ground-truth image than those obtained by the *k*-space and OMP methods, thus verifying the performance superiority of the PROMP method.

Table 2 compares the runtimes of the *k*-space, OMP, and PROMP algorithm for the image results shown in Figure 4b. For a fair comparison, all methods were implemented in MATLAB on the same Intel Core i7 3.40 GHz CPU with 16 GHz RAM. We observe that the *k*-space method is much slower than the OMP and PROMP methods. The reason for this is that the *k*-space method requires the non-uniform fast Fourier transform to be performed, thus being computationally more demanding compared to OMP and PROMP. On the other hand, the OMP and PROMP methods are computationally fast, thanks to the fact that they belong to the greedy pursuit family. In addition, each iteration of PROMP is about 7.3 times slower than each iteration of OMP because PROMP incorporates a NLS solver, rather than a linear least-squares solver as in OMP. However, the overall timerun of PROMP is only 2.4 times slower than that of OMP because PROMP requires many fewer iterations than OMP to make the signal residual reach the noise level.

### 5.2. Results with Real Data

The experimental data used in this paper was collected in the Mumma Radar Laboratory at the University of Dayton, Ohio, USA. Although this paper focuses on narrowband tomographic imaging, a wideband waveform at X-band with stepped frequency pulses over 101 regular frequency steps from 8 GHz to 12 GHz was used in the experiment. The aim of using wideband data was only for the removal of extraneous clutter components existing in the lab environment, as described in [13]. After that, only the measured data from one discrete frequency is actually used for algorithm performance evaluation for the problem of narrowband tomographic imaging under consideration.

**Figure 7:**
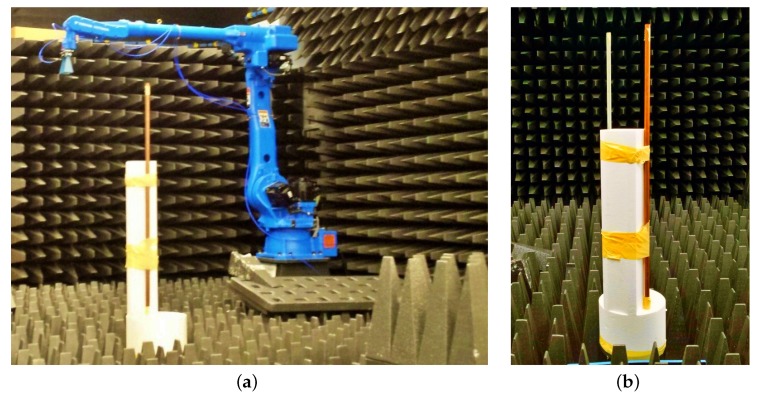
Photos of experimental system configuration: (**a**) an antenna mounted on a robotic arm, and (**b**) two vertical metallic rods secured to a rotating pedestal.

**Figure 8:**
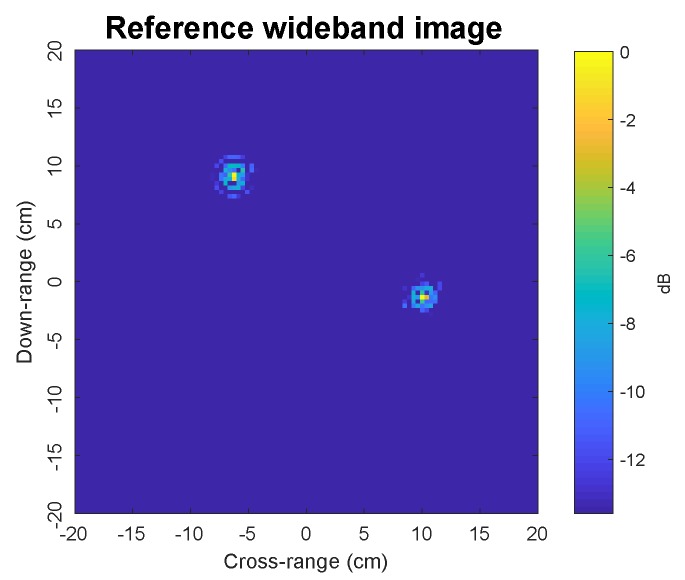
A benchmark experimental target image using wideband signals received by the monostatic receiver for all 101 frequency steps between 8 GHz and 12 GHz.

Figure 7 shows photos of the experimental system configuration. The experimental setup involves transmitting and receiving horn antennas mounted on separate robotic arms in a controlled laboratory environment with radar-absorbing material to reduce the radar reflections from the floor and walls. These robotic arms could be oriented and positioned with high precision. During the experiment, the antennas were kept stationary while the target was rotated over $1^{\circ }$ steps through $360^{\circ }$, where the stepped-frequency waveform was transmitted and sampled (one sample for each frequency). Recall from above that only one frequency sample set was used for tomographic imaging purposes. The experimental target was comprised of two vertical metallic rods with a 19 cm separation to emulate two point scatterers which rotate around a vertical pedestal. Figure 8 shows the wideband *k*-space tomographic image obtained from monostatic data with all 101 frequency steps. This will be used as a reference benchmark for the performance evaluation of narrowband bistatic imaging presented in this section.

**Figure 9:**
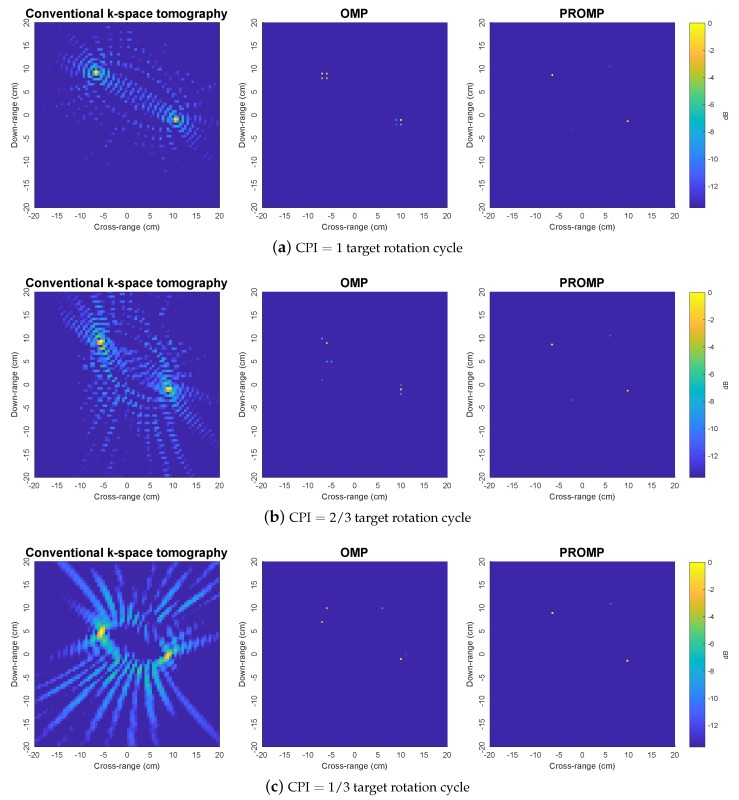
Experimental narrowband images obtained by the *k*-space, OMP, and PROMP algorithms using a narrowband signal received by the bistatic receiver with $\beta =86^{\circ }$ at a single frequency of 8.8 GHz, for various values of CPI.

Figure 9 shows the experimental narrowband images obtained by the *k*-space, OMP, and PROMP algorithms using a narrowband signal received by the bistatic receiver with $\beta =86^{\circ }$ at a single frequency of 8.8 GHz for various values of CPI. Since the SNR is unknown, the OMP and PROMP iterations are halted when the change in the signal residual norm falls below 1% of the input signal norm. Compared to the benchmark image in Figure 8, the *k*-space method produces images with a much lower quality when only a narrowband signal from a single frequency is available. We observe that the images obtained by the *k*-space method are distorted with numerous spurious sibelode peaks. In particular, the number of spurious sibelode peaks increases significantly for shorter CPI. Moreover, the main lodes corresponding to the true scatterers are also spread when the CPI is reduced. In contrast, the PROMP images contain two clear peaks at the locations very close to the peaks of the reference image in Figure 8, even when the CPI is reduced to one third of the target rotation cycle. This observation demonstrates the performance superiority of the sparsity-based tomography approach over the conventional *k*-space tomography approach. Figure 9 also shows the images obtained by OMP to illustrate the dictionary mismatch problem associated with off-grid scatterers, where each true scatterer is split into multiple peaks; thus, verifying the effectiveness of PROMP in terms of tackling the dictionary mismatch problem.

**Figure 10:**
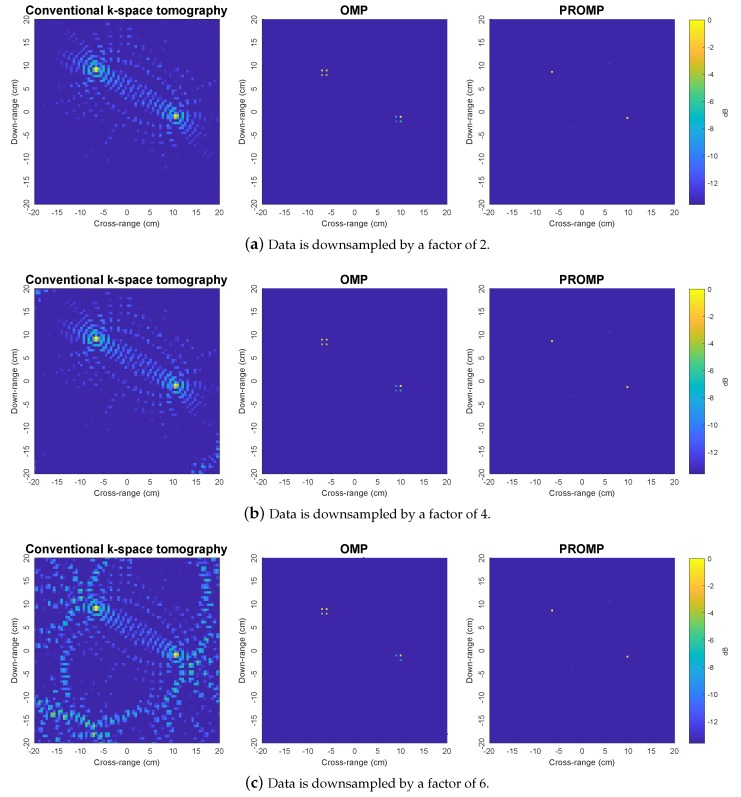
Experimental narrowband images obtained by the *k*-space, OMP, and PROMP algorithms for downsampled data (CPI=1 target rotation cycle).

Figure 10 shows the experimental results where the data is downsampled. Here, we observe a similar relative performance comparison to Figure 3, Figure 4 and Figure 5 and Figure 9, once again confirming the performance advantages of the proposed sparsity-based tomographic imaging method based on the PROMP algorithm.

## 6. Conclusions

In this paper, we have proposed a new sparsity-based bistatic radar tomographic imaging method exploiting the use of the PROMP algorithm. A new formulation for radar tomography building on the framework of compressive sensing and sparse reconstruction was presented, moving away from conventional *k*-space tomography which is prone to sidelobe responses and their interference. The PROMP algorithm was adopted to obtain a sparse solution for the resulting sparsity-based tomography formulation. By perturbing the dictionary atoms and allowing the estimated scatterers to go off the grid, PROMP is capable of tackling the dictionary mismatch problem arising from off-grid scatterers. The performance advantages of the proposed sparsity-based tomography method over the conventional *k*-space tomography method were demonstrated via numerical studies involving both simulated and real data.

## Figures and Tables

**Table 1:** Summary of PROMP.

INPUT: $\tilde{s},\Phi$.
Initialization: $r^{\left[0\right]}=\tilde{s}$, $\Lambda^{\left[0\right]}=\emptyset$ **for** iteration i=1; i:=i+1 until stopping criterion is met **do** - Identify: $c^{\left[i\right]}=\Phi^{H}r^{\left[i-1\right]}$ $j^{\left[i\right]}={arg max}_{j}|c_{j}^{\left[i\right]}|$ quit the iteration **if** $j^{\left[i\right]}\in \Lambda^{\left[i-1\right]}$ - Record selected supports: $\Lambda^{\left[i\right]}=\Lambda^{\left[i-1\right]}\cup j^{\left[i\right]}$ - Nonlinear least-squares estimation: $$\begin{array}{c} \;\;{\left\{{\hat{\sigma }}_{k}^{\left[i\right]},{\hat{x}}_{k}^{\left[i\right]}\right\}}_{k=1,\ldots ,i}=arg min∥\tilde{s}-\sum_{k=1}^{i}\sigma_{k}^{\left[i\right]}\phi \left(x_{k}^{\left[i\right]}\right){∥}_{2}\\ \;\mathrm{subject}\;\mathrm{to}\\ \;\;∥{\hat{x}}_{1,k}^{\left[i\right]}-{\bar{x}}_{1,k}^{\left[i\right]}∥\leq \zeta \;\mathrm{and}\;∥{\hat{x}}_{2,k}^{\left[i\right]}-{\bar{x}}_{2,k}^{\left[i\right]}∥\leq \zeta \end{array}$$ - Update residual: $r^{\left[i\right]}=\tilde{s}-\sum_{k=1}^{i}{\hat{\sigma }}_{k}^{\left[i\right]}\phi \left({\hat{x}}_{k}^{\left[i\right]}\right)$
**end for**.
OUTPUT: ${\left\{{\hat{\sigma }}_{k}^{\left[i\right]},{\hat{x}}_{k}^{\left[i\right]}\right\}}_{k=1,\ldots ,i}$

The superscript ^*H*^ stands for the Hermitian transpose.

**Table 2:** Complexity comparison ${}^{⋆}$.

Algorithm	*k*-Space	OMP	PROMP
Averaged runtime (second)	0.1561	0.0331	0.0799
Averaged number of iterations	−	27.65	8.96
Averaged per-iteration runtime (second)	−	0.0012	0.0088

${}^{⋆}$ All methods are implemented in MATLAB on an Intel Core i7 3.40 GHz CPU with 16 GHz RAM, for the image results shown in Figure 4b.
